# Derivation of machine learning brain aging biomarkers for a set of forty thousand functional connectomes

**DOI:** 10.1016/j.brainresbull.2026.111815

**Published:** 2026-03-05

**Authors:** Nicolas Honnorat, Di Wang, Ngoc-Huynh Ho, David Martinez, Sachintha Ransara Brandigampala, Susan R. Heckbert, Mohsen Bahrami, Jayandra Jung Himali, Charlie DeCarli, Alexa Beiser, Timothy M. Hughes, Sudha Seshadri, Mohamad Habes

**Affiliations:** a Glenn Biggs Institute for Alzheimer’s & Neurodegenerative Disease, University of Texas Health Science Center at San Antonio, 4940 Charles Katz Drive, San Antonio, 78229, TX, USA; b Section of Gerontology and Geriatric Medicine Department, Wake Forest University School of Medicine, Medical Center Boulevard, Winston-Salem, 27157, NC, USA; c Department of Epidemiology, University of Washington School of Public Health, 3980 15th Ave NE, Box 351621, Seattle, 98195, WA, USA; d Department of Neurology, University of California Davis, 1651 Alhambra Blvd Suite 200A, Sacramento, 95816, CA, USA; e Department of Biostatistics, Boston University School of Public Health, 715 Albany Street, Boston, 02118, MA, USA

**Keywords:** Functional MRI, Aging

## Abstract

Various Magnetic Resonance Imaging modalities were developed to explore the brain. Among them, functional MRI is of key importance for studying brain activity and its neural substrates. Recent works have pointed out that machine learning can use neuroimaging data to predict brain age. This approach is crucial not only for understanding the effects of aging but also for refining diagnostics because many chronic and neurodegenerative diseases appear as accelerated aging. Unfortunately, the prediction of brain age is particularly challenging for functional data due to the large dimension of the high-resolution connectomes usually derived to summarize the functional organization of the brain and their particular mathematical properties. In this work, we investigate the prediction of brain age from functional data on a large scale by creating a set of forty thousand functional connectomes via the processing of the resting-state fMRI scans of four cohort studies. This dataset is used to explore the ability of various connectome transformations and machine learning strategies to achieve accurate age predictions. We hope that our results will open the way for more reliable functional brain age measures.

## 1. Introduction

Magnetic Resonance Imaging (MRI) is a prominent neuroimaging tool. Structural modalities, such as T1-weighted and T2-weighted MRI scans, can produce extremely detailed and valuable 3D images of the brain, while functional MRI is used to record brain activity (Fox and Raichle, 2007). The activity recorded during resting-state fMRI scans, a type of scan in which brain activity is captured for several minutes while study participants focus on no particular mental task (Birn et al., 2013), provides crucial information about the internal organization of the brain (Fox and Raichle, 2007; Smith et al., 2011). The scans are usually analyzed by calculating the correlation between the activity recorded in different brain locations (Fox and Raichle, 2007; Smith et al., 2011; Bullmore and Sporns, 2009; Rubinov and Sporns, 2010). Highly correlated or strongly anti-correlated activities indicate that two brain regions are interacting, and this association is known as functional connectivity (Smith et al., 2011; Fox and Raichle, 2007). When a map segmenting the brain into regions of interest is used as a reference, the functional connectivity between all pairs of regions can be summarized in a graph: the functional connectome (Bullmore and Sporns, 2009; Rubinov and Sporns, 2010), and it becomes possible to group highly connected regions into functional networks (Yeo et al., 2011; Buckner et al., 2008). Functional connectomes and functional networks change during life (Cole, 2020). They reflect individual cognitive (Zhu et al., 2021) and personality traits (Cai et al., 2020b,a), and they are altered by multiple neurodegenerative (Wang et al., 2006; Dennis and Thompson, 2014; de Vos et al., 2018) and psychiatric disorders (Mwansisya et al., 2017; Sabbah and Northoff, 2024; Craddock et al., 2013; Yang et al., 2018). As a result, fMRI can be used to derive neuroimaging biomarkers (Craddock et al., 2013; Bullmore and Sporns, 2009; Rubinov and Sporns, 2010), and statistical and machine learning models can be developed to associate functional connectome variations with individual and cognitive characteristics, such as personality (Cai et al., 2020b), working memory (Zhu et al., 2021), and decision impulsivity (Cai et al., 2020a). A recent trend of work has focused on a specific way of deriving such neuroimaging biomarkers: the prediction of age from MRI scans (Cole et al., 2017, 2018). This approach is crucial for studying brain aging and offers a way of improving brain disorder diagnosis because many diseases appear as accelerated aging (Cole et al., 2017, 2018; Cole, 2020; Moguilner et al., 2024). Machine learning can therefore be used to define *brain clocks* that reflect the health of specific brain structures (Hou et al., 2019; Tian et al., 2023; Moguilner et al., 2024). Brain age is usually more challenging to estimate from functional data than structural MRI scans because reliable connectomes are difficult to estimate (Smith et al., 2011; Honnorat and Habes, 2022), but an increasing set of literature is exploring this possibility (Cole et al., 2017, 2018; Cole, 2020; Moguilner et al., 2024) with the hope of developing functional aging biomarkers that could expand the structural markers (Eavani et al., 2018; Cole et al., 2017, 2018).

In this work, we focus on this more challenging setting by exploring the use of advanced statistical frameworks and recent preprocessing methods to achieve better age predictions and produce more reliable metrics of functional brain aging. More specifically, we create a large set of more than forty thousand functional connectomes by processing the resting-state fMRI scans of four cohort studies: the Framingham Heart Study (FHS (Debette et al., 2010; Zhao et al., 2024)), the Human Connectome Project (HCP (Van Essen et al., 2013)), the Multi-Ethnic Study of Atherosclerosis (MESA) (Bild et al., 2002; Burke et al., 2016; Olson et al., 2016), and the UK Biobank (UKBB) (Miller et al., 2016; Alfaro-Almagro et al., 2018; Sudlow et al., 2015). This large data set is used to compare three functional connectome transformations and three machine learning methods to achieve accurate age predictions. For the best age predictor, the difference between the model-predicted age and the actual age of the study participants is used to define a functional aging biomarker. We demonstrate that this functional biomarker is significantly correlated with many health and cognitive markers and postulate that it may reflect brain health.

## 2. Materials and methods

**Fig. 1.**
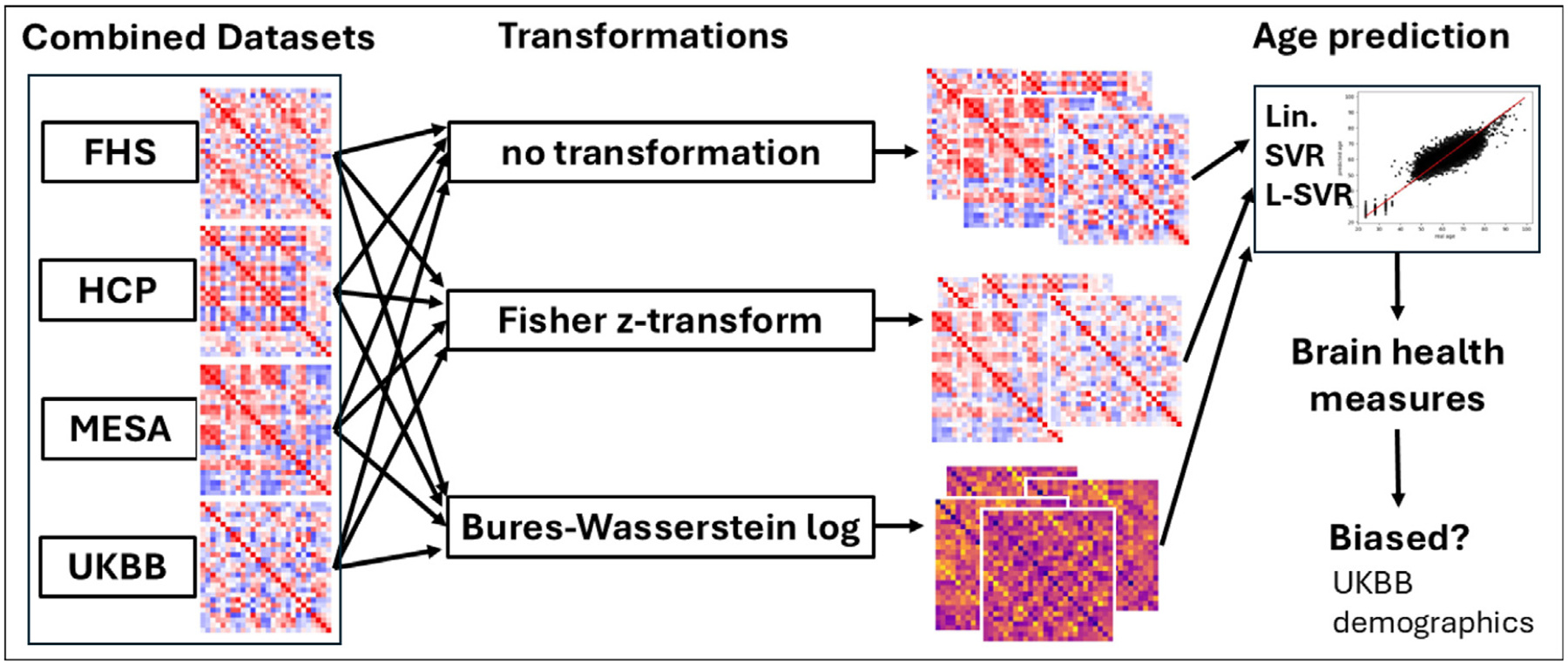
Overview: the functional connectomes derived from the rs-fMRI scans of four cohort studies were combined, transformed, and used to build age predictors. Brain aging biomarkers were derived from the best cross-validated models, and statistical tests were conducted using the UKBB metadata to investigate the presence of biases in these measures.

This Section presents the four datasets combined during our experiments, describes the age predictors, and indicates how the models were trained and validated. Our functional aging biomarker and the investigations carried out to reduce its biases are presented at the end of the Section. The overall approach is summarized in Fig. 1.

### 2.1. Data

This work combines the resting-state fMRI scans of four large cohort studies: the Framingham Heart Study (FHS) (Debette et al., 2010; Zhao et al., 2024), the Human Connectome Project (HCP) (Van Essen et al., 2013), the Multi-Ethnic Study of Atherosclerosis (MESA) (Bild et al., 2002; Burke et al., 2016; Olson et al., 2016), and the UK Biobank (UKBB) (Miller et al., 2016; Alfaro-Almagro et al., 2018; Sudlow et al., 2015). FHS data access procedures can be obtained here: https://www.framinghamheartstudy.org/fhs-for-researchers/. The Human Connectome Project minimally preprocessed young adult dataset imaging data and associated NIH Toolbox measures are publicly available at https://db.humanconnectome.org/. The MESA data was processed with the approval of the MESA Coordinating Center. The instructions for accessing the MRI scans are provided here: https://www.mesa-nhlbi.org/. The UKBB data mentioned in this work is available to all researchers and can be accessed upon approval of the UK Biobank (https://www.ukbiobank.ac.uk/enable-your-research/apply-for-access).

Overall, the resting-state fMRI scans of 413 FHS participants were included, as well as the scans of 1015 HCP participants, 1057 MESA participants, and 37929 UKBB participants. The demographics of these 40414 study participants are summarized in Table 1. A set of statistical tests were conducted using R, version 4.1.1 (R Core Team, 2021) to compare the four cohorts. The four Fisher exact tests conducted to compare the proportions of men and women within each study with the proportion of men and women in the entire sample detected no statistically significant difference at level p<0.05. On the other hand, the two sample T-tests carried out to compare the mean age of men and women within each study group detected two very significant differences (at level p<0.001): the women in the HCP sample were on average 1.39 years older than the HCP men, and the men in the UKBB samples were 1.3 years older than the UKBB women. As a result, in the overall sample of 40414 participants, men were, on average, 1.19 years older, and that difference was also very significant (at level p<0.001). Cohen’s D was measured to assess the effect size associated with these significant statistical differences (Cohen, 1988). The largest effect size, of value 0.396, was observed for the HCP and was small, as well as the effect sizes of 0.172 measured for the UKBB and 0.126 for the entire data set (Cohen, 1988). The same statistical tests were carried out to compare the mean age recorded for all the participants of a single study with the mean age calculated for the entire set of participants. This time, all the statistical differences were very significant: FHS and HCP participants were younger than the average, and MESA and UKBB participants were older than average. A small effect size was observed for the UKBB (Cohen’s D 0.172), but the effect size was medium for FHS (Cohen’s D 0.491) and large for MESA (1.181) and the HCP sample (Cohen’s D 3.649).

In conclusion, the four cohorts present similar proportions of men and women. The age of study participants can sometimes be biased by their sex, but these biases are small. The main age biases appear when comparing studies and noticing that they cover significantly different age groups ranging from young adults for the HCP to elderly study participants in MESA.

### 2.2. Processing

#### 2.2.1. HCP

The Human Connectome Project provides preprocessed resting-state fMRI data (Van Essen et al., 2013). In preparation for this work, the entire set of fully processed scans of the HCP_1200 release was downloaded. It was possible to recover demographic information (age and sex) for 1015 HCP study participants with a complete set of four processed scans, indicated in the HCP repository with the following file extensions (Van Essen et al., 2013):


 _REST1\_LR\…\_clean.dtseries.nii
 _REST1\_RL\…\_clean.dtseries.nii
 _REST2\_LR\…\_clean.dtseries.nii
 _REST2\_RL\…\_clean.dtseries.nii
where … replaces _Atlas\_MSMAll\_hp2000


**Fig. 2.**
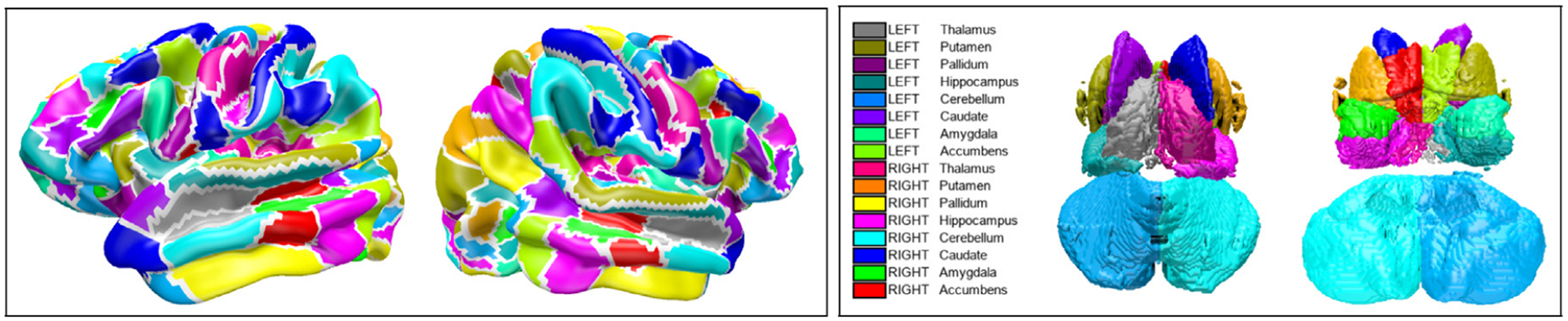
Left: 360 cortical parcels of the Conte69 atlas (left picture: left hemisphere, right picture: right hemisphere). Right: 16 deep grey matter regions of the Conte69 atlas (left picture: superior view, right picture: inferior view).

The four scans of these 1015 study participants were processed independently as follows. Each scan was downloaded, detrended, and a temporal Butterworth bandpass filter of order 2 with a sampling frequency of 1.0/0.7275 was applied to retain rs-fMRI temporal frequencies between 0.01 and 0.1 Hz. This sampling frequency was selected because the volumes of the HCP rs-fMRI scans were acquired every 727.5 ms (Van Essen et al., 2013). For each time point, the median of the rs-fMRI signal was calculated for the cortical parcels (180 parcels) and for eight of the deep grey matter regions defined in the HCP parcellation: the nucleus accumbens, the amygdala, the caudate, the cerebellum, the hippocampus, the globus pallidus, the putamen, and the thalamus (Glasser et al., 2016). The regions defined in the HCP parcellation (Glasser et al., 2016) are shown in Fig. 2. This operation was conducted separately for both hemispheres, producing 376 regional time series. Global signal regression was conducted, and the time series were normalized to zero mean and unit L2 norm (Honnorat and Habes, 2022; Honnorat et al., 2024). At this step, the time series of the four scans available for each study participant were concatenated, the concatenated time series were normalized again to a unit L2 norm, and a connectome was calculated by computing the Pearson correlations between the time series and applying the Oracle Approximating Shrinkage (Honnorat and Habes, 2022; Chen et al., 2010). The Oracle Approximating Shrinkage is known to improve the test-retest reliability of functional connectomes by producing correlation matrices that are closer to the “true” correlation values that would be derived from infinitely long fMRI scans than the original Pearson correlations typically used in fMRI connectivity analysis (Honnorat and Habes, 2022).

#### 2.2.2. FHS and MESA

The neuroimaging data of the 413 FHS and the 1057 MESA study participants were processed from scratch (Debette et al., 2010; Zhao et al., 2024; Bild et al., 2002; Burke et al., 2016; Olson et al., 2016) by using the image processing pipeline developed during our prior work (Honnorat et al., 2024). This pipeline requires a T1-weighted structural MRI scan to process a rs-fMRI scan. The structural T1-weighted scans are processed at first. N4 bias field correction (Tustison et al., 2010) and NAONLM3D (Manjón et al., 2010) are applied to denoise the scan. ANTS (version 2.1.0-gGIT-N) SyN registration (Avants et al., 2011, 2008) is then used to register the denoised T1-weighted scan to the HCP atlas (Van Essen et al., 2012), and the atlas brain mask is warped into the T1-weighted scan to mask the structures outside the brain. The resting-state scans are then processed by using FSL MCFLIRT (FSL 5) to conduct motion correction and fslmath to average the fMRI signal over time. FSL BET is applied to that average to produce a brain mask that is used to remove the resting-state fMRI signal outside the brain (Jenkinson et al., 2012). FSL epi_reg is then used to warp each fMRI scan into the space of the corresponding processed T1-weighted scan, and the ANTS transformation obtained when processing that structural scan is applied to warp the scan in the HCP reference space (Avants et al., 2011; Van Essen et al., 2012), where advanced signal processing is performed. More specifically, outlier fMRI volumes are first detected by investigating the displacements recorded by FSL MCFLIRT and searching for either (1) a change in signal intensity of more than three standard deviations from the mean intensity or (2) a displacement larger than 3 mm between MRI volumes (Power et al., 2014). FSL GLM (Friston et al., 1994) is then used to remove and interpolate these corrupted volumes while removing fourteen additional temporal time series from the rs-fMRI time series: six time series derived from the recorded motion (the motion, its square, their derivatives, and the square of their derivatives), five time series corresponding to the most prominent Singular Value Decomposition components of the fMRI signal measured in the HCP atlas white matter, a constant intercept, a linear and a quadratic time trends modeled using Legendre polynomials. At this point, the band-pass filtering described in the previous Sections was applied, followed by a spatial smoothing of 4 mm standard deviation conducted using FSL (fslmath -s command). The connectome workbench (version 1.5.0) was used to project the data, and a connectome was derived from the projected time series in the same way as for the preprocessed HCP scans.

#### 2.2.3. UKBB

For the UK Biobank, 37,929 study participants with fully processed fMRI data and demographics could be recovered (Miller et al., 2016; Alfaro-Almagro et al., 2018; Sudlow et al., 2015). For these study participants, two ICA time series were available and downloaded: a set of 25 ICA time series and a set of 100 ICA time series. For each ICA, the 37,929 individual fMRI scans were reconstructed by multiplying the ICA spatial maps by the ICA temporal loadings. The connectome workbench (version 1.5.0) (Van Essen et al., 2012; Marcus et al., 2011) was then used to project these 4D time series within the cortical and deep grey matter regions defined in the HCP parcellation (Glasser et al., 2016). The two sets of regional time series were averaged, and the average time series were processed like HCP time series by applying bandpass filtering, global signal regression, normalization, calculating a connectome, and using the Oracle Approximating Shrinkage to improve that connectome (Honnorat and Habes, 2022; Chen et al., 2010). As a result, compared to FHS, HCP, and MESA, our UKBB regional time series and connectomes were simply derived from ICA-denoised time series rather than from time series denoised using a spatial Gaussian filter. The derivation of connectomes after ICA denoising is a very common approach in the field, for instance, to validate pipelines (Wang et al., 2024) and ICA methods (Pruim et al., 2015a). Well-established ICA methods, such as ICA-AROMA (Pruim et al., 2015b), were validated in this way (Pruim et al., 2015a), and this strategy is still adopted in the most recent literature (Goffi et al., 2025). However, the different preprocessing steps conducted across our four clinical cohorts might have introduced slight biases. They were mitigated by applying a state-of-the-art harmonization method (Fortin and Cullen, last checked in Nov. 2025), as explained in Section 2.3.3. Supplementary Section 4 reports additional experiments demonstrating the effectiveness of our harmonization procedure.

### 2.3. Age predictors

This work compares a large variety of age predictors. More specifically, three different transforms were applied to the connectomes to improve their statistical distributions. A truncated Singular Value Decomposition (SVD) was then used to retain the most relevant variability in the connectomes. Harmonization was performed, and the data were split into training and test sets. Three types of age regressors were then applied: linear regressors regularized by an L2 squared penalty, also known as ridge regressors, Linear Support Vector Regressors, and nonlinear Support Vector regressors relying on a Radial Basis Function kernel (Drucker et al., 1996; Cortes, 1995; Smola and Schölkopf, 2004). These steps are detailed in the following Sections.

#### 2.3.1. Connectome transforms

Let $C_{s}$ denote the connectome calculated for the study participant s, and $C_{s,ij}$ the functional connectivity between the regions i and j defined in the HCP parcellation for that study participant s (Glasser et al., 2016). The first age predictors tested in this work were trained to directly predict the age of the study participant s from the truncated SVD of the $C_{s}$. The second group of age predictors was trained to predict the age of s from the truncated SVD of the Fisher z-transform (Clayton Silver and Dunlap, 1987) $D_{s}$ of $C_{s}$. $D_{s}$ was calculated to avoid divisions by zero:

(1) $$D_{s,ij}=\frac{1}{2}\text{log}\;\left(\frac{1+\mu C_{s,ij}}{1-\mu C_{s,ij}}\right)$$

(2) whereμ=0.999

The last group of age predictors was trained after replacing the connectome $C_{s}$ with the Bures–Wasserstein log matrix $E_{s}$ derived according to our previous work (Honnorat et al., 2024):

(3) $$E_{s}={\left(BC_{s}\right)}^{1/2}+{\left(C_{s}B\right)}^{1/2}-2B$$

where B denotes the reference connectome where the log matrix is calculated, and the calculation relies on matrix square roots instead of element-wise operations (Honnorat et al., 2024). Since we have established in our previous work (Honnorat et al., 2024) that the choice of the reference connectome B for the calculation of a Bures–Wasserstein log matrix has a negligible impact on downstream regression tasks, a change of reference matrix mostly acting as a data shift, the reference connectome was set to be the identity matrix I to simplify and accelerate the calculations:

(4) $$E_{s}=2\left(C_{s}^{1/2}-I\right)$$

#### 2.3.2. Truncated SVD

The connectomes $C_{s},\;D_{s}$, and $E_{s}$ are of size 376 × 376, but they are symmetric. As a result, their upper triangular parts including the diagonal are sufficient to completely describe an individual connectome. These upper parts were flattened into rows of dimensions 1 × 70876 that were stacked to create a data matrix A of size 40414 × 70876 for each type of connectomes. A Singular Value Decomposition (SVD) was then conducted:

(5) A=USV

and the sum of the squares of the diagonal components of S was calculated. The rows of V corresponding to the largest singular values in S and with the smallest total sum of squares larger than a proportion π of the original sum were retained to create a matrix W of size k×70876, which was used to compress the data:

(6) $$\overset{‾}{A}=AW^{T}$$

into thinner matrices, of size 40414×k with k≪70876. During our experiments, the proportion π was set to 99.9%. This proportion π produced data matrices $\overset{‾}{A}$ with less than five thousand columns, much thinner than the original flattened connectomes matrices A, and capturing the most important variability in the training connectomes A.

#### 2.3.3. Harmonization and split

Combining MRI scans acquired in different scanners, using different neuroimaging protocols, or preprocessed with different pipelines can introduce biases during statistical analysis. Harmonization methods were developed to mitigate this issue (Johnson et al., 2006; Fortin and Cullen, last checked in Nov. 2025; Honnorat et al., 2024). Following our prior work (Honnorat et al., 2024) and state-of-the-art standards (Fortin and Cullen, last checked in Nov. 2025), the neuroCombat method (Johnson et al., 2006; Fortin and Cullen, last checked in Nov. 2025) was applied to the data obtained after SVD truncation $\overset{‾}{A}$ while considering the age at scan and the sex as covariates (Johnson et al., 2006). The rows of the harmonized matrix were then split to separate the training samples in a matrix $\overset{‾}{X}$ and the test scans in a matrix $\overset{‾}{Z}$ as explained in Section 2.4 below. Let denote with $n_{x}$ the number of rows retained in $\overset{‾}{X}$ and $n_{z}$ the number of rows in $\overset{‾}{Z}$.

#### 2.3.4. Age regressors

The ages of the study participants were stacked in vectors $Y_{x}$ and $Y_{z}$ of size $n_{x}\times 1$ and $n_{z}\times 1$, and the age regressors were trained to predict $Y_{x}$ from $\overset{‾}{X}$. The age predictors were tested by predicting a vector $Y_{p}$ from the compressed connectomes $\overset{‾}{Z}$ of the test participants. $Y_{p}$ was compared with the correct test participant ages $Y_{z}$ to measure the performance of the age predictors. Specifically, the mean absolute error (MAE) between the predicted ages $Y_{p}$ and the real ages $Y_{z}$ was calculated:

(7) $$MAE=\frac{1}{n_{z}}\sum_{s}\left|Y_{p}(s)-Y_{z}(s)\right|$$

where $Y_{p}(s)$ and $Y_{z}(s)$ denote the predicted and the real ages of the test study participant s.

#### 2.3.5. Ridge regression

The first age predictors tested in this work correspond to ridge regressors. To model a linear intercept, a column of ones was added at the beginning of both matrices $\overset{‾}{X}$ and $\overset{‾}{Z}$ to create the matrices $\underline{X}$ and $\underline{Z}$ that were used to calculate the linear weights β of the ridge regressors and generate the prediction $Y_{p}$ for the study participants in Z as follows:

(8) $$\beta ={\left({\underline{X}}^{T}\underline{X}+\lambda I\right)}^{-1}{\underline{X}}^{T}Y$$

(9) $$Y_{p}=\underline{Z}\beta$$

where λ denotes the ridge penalty. The parameter λ was set by cross-validation during our experiments.

#### 2.3.6. Support vector regressors

More sophisticated age predictors were tested by training Support Vector Regressors (SVR, Drucker et al. (1996)), a variant of the Support Vector Machines (Cortes, 1995; Smola and Schölkopf, 2004) binary classifier that produces a continuous output instead of a binary classification (Cortes, 1995; Smola and Schölkopf, 2004; Drucker et al., 1996). Two different support vector regressors were compared in this work: linear SVRs relying on a linear Mercer kernel and SVMs relying on a radial basis function (RBF) Mercer kernel (Cortes, 1995; Smola and Schölkopf, 2004). The scikit-learn Python library was used to implement these age regressors (Pedregosa et al., 2011). The Scikit-learn SVR code relies on the efficient LIBSVM and LIBLINEAR software (Fan et al., 2008). For both types of SVR, the C-SVR variant was selected, and the parameter C was set by cross-validation during our experiments (Cortes, 1995; Smola and Schölkopf, 2004; Drucker et al., 1996).

### 2.4. Models training and validation

#### 2.4.1. Stratification

The age prediction was improved by fitting separate models for men and women. With more than forty thousand connectomes and a good balance between men and women, respectively corresponding to 47% and 53% of the sample size, this sex stratification was the most straightforward strategy to account for potential sex biases in our neuroimaging data. The approach worked well during our experiments. A stratification was also conducted based on the neuroimaging study that provided the scans to ensure that the same proportions of FHS, HCP, MESA, and UKBB scans were used when cross-validating, training, and testing our age predictors. This stratification strategy ensures that the variability across datasets in imaging protocols and preprocessing has affected all age predictors equally and has not affected method comparisons. The stratification is presented in detail in Table 2.

#### 2.4.2. Parameter settings and validation

For each sex group, a stratified random split was carried out to separate the entire list of connectomes in two data sets: a small set (**parameter setting** set) where a Monte-Carlo cross-validation was conducted to select the ridge penalties λ, and the C parameters of the age predictors, and a large set (**method validation** set) gathering the remaining scans and where a ten-fold cross-validation was conducted, using the proportions and parameter values selected at the previous stage, to measure a final set of MAE values and specifically investigate the most recent approach tested in this work: the use of a Bures–Wasserstein transform instead of a Fisher z-transform to modify connectivity values prior to their compression.

More specifically, separately for each sex group, the scans of each cohort were randomly shuffled, the first ten percent of scans in each study were separated, the small groups obtained for the four studies were combined to create a parameter setting data set for each sex, and the remaining scans were combined to create one method validation set for each sex. These breakdowns are shown in Table 2. Overall, parameter setting was conducted using 1895 scans for the age predictors created to predict men ages and 2144 for women age predictors, while the final method validation was carried out using 17070 scans in men and 19305 scans in women.

A Monte Carlo cross-validation was conducted during the parameter setting (Kuhn and Johnson, 2013). Twenty-five times in a row, the scans were shuffled and separated into two halves: the first half was used to train the age predictors and the second half was used to measure their performance by calculating the mean absolute error (MAE) between the predicted age and the real study participant age. This cross-validation approach, more reliable but also more time-consuming than a standard ten-fold cross-validation, could be conducted for all the connectome transforms. For the ridge regressors, twelve ridge penalty values were tested: 0, 10, 25, 50, 75, 100, 250, 500, 750, 1000, 2500, 5000. For the linear SVR regressors, nine values were considered for the inverse of the C parameter 10, 25, 50, 75, 100, 250, 500, 750, 1000, and for the SVR regressors relying on a RBF kernel, the inverse of C was selected among a set of nine values: 0.001, 0.0025, 0.005, 0.0075, 0.01, 0.025, 0.05, 0.075, and 0.1.

The final method validation results were obtained via a ten-fold cross-validation: the method validation data set was separated into ten parts, and for each part, the age predictors were trained using the remaining scans and tested using the scans in the part. A mean absolute error was obtained for each part, and the ten MAEs obtained for each method were compared with the values obtained for the other methods. During this method validation, the ridge penalty λ, and the C parameters were set according to the best results obtained during the parameter setting experiments. As a result, only nine methods were compared for each sex group: age predictors relying on ridge regressors, SVRs or Linear SVRs, derived for the original correlation matrices, Fisher z-transformed connectomes, or Bures–Wasserstein log-correlation. Since the same data folds were used to train age predictors relying on different methods during the Monte Carlo or the ten-fold cross-validations, the mean absolute errors could be directly compared, and we could conduct Wilcoxon signed-rank tests to estimate a level of statistical significance when a method exhibited smaller age prediction errors (Conover, 1999).

Because the nonlinear SVR required too much training time during the method validation experiments, the SVR method validation experiments were conducted with a random tenth of the prepared training data. Linear SVR and ridge regressors could be trained using the full training dataset. The three methods were evaluated using the same complete test data.

### 2.5. Brain aging biomarkers

The difference δ between the age predicted from fMRI scans by machine learning models and the real age of a participant in a neuroimaging study is expected to reflect the accelerated aging of the brain associated with a diseased condition. This δ is potentially associated with age, so we defined our functional brain aging biomarkers by computing the opposite of a residual obtained after removing a linear age trend:

(10) $$\mu =-\left(\delta -\delta^{*}\right)$$

(11) $$\delta^{*}=\beta_{1}+\beta_{2}\text{age}+\beta_{3}\text{sex}+\beta_{4}\text{.age.sex}$$

and $\delta^{*}$ was fitted to approximate δ. The linear coefficients $\beta_{1},\beta_{2},\beta_{3},\beta_{4}$ were calculated by fitting an ordinary least square model (OLS) with the Python statsmodels API (version 0.12.2). The measure μ was defined with a flipped sign relative to δ to obtain a positive measure when the brain appears younger than it should be, assuming that positive values might be associated with higher health biomarkers. The associations between the brain aging biomarkers μ derived from the best age prediction models for the UK Biobank (Miller et al., 2016; Alfaro-Almagro et al., 2018; Sudlow et al., 2015) and a large set of biomarkers reported by this study were estimated by fitting the following OLS and reporting the p-value associated with the coefficient α, the adjusted $R^{2}$ of the OLS model, the standardized coefficient β and its 95% confidence interval:

(12) μ≈cst.+α.biomarker

(13) $$\beta =\frac{\text{std}(\mu )}{\text{std}(\text{biomarker})}\alpha$$

The standardized coefficient β is equal to the Pearson correlation between the biomarker values and the μ values. A set of sixteen biomarkers was considered. Four measures reflecting the overall health of the study participants were included: the body mass index (BMI, UKBB field #21001.0.0), the number of non-cancer illnesses (#135.0.0), the number of ongoing medical treatments (#137.0.0), and the overall health (#2178.0.0, encoded as 0 for “poor”, 1 for “fair”, 2 for “good”, 3 for “excellent”). In addition, four cardiovascular measures were considered: automatically recorded systolic (#4080.0.0) and diastolic blood pressures (#4079.0.0), cardiac pulse rate (#102.0.0), and arterial stiffness index. The arterial stiffness index was calculated by dividing the height of the study participant (#12144.2.0) by the pulse propagation time reported by the UKBB (#4196.0.0) (Said et al., 2018). This set of biomarkers was completed with three potential risk factors: alcohol intake frequency (#1558.0.0 encoded as 5 for “Daily or almost daily”, 4 for “Three or four times a week”, 3 for “Once or twice a week”, 2 for “One to three times a month”, 1 for “Special occasions only”, and 0 for “Never”), processed meat intake (#1349.0.0 encoded as 0 for ”Never“, 1 for “Less than once a week”, 2 for “Once a week”, 3 for “2–4 times a week”, 4 for “5–6 times a week”, 5 for “Once or more daily”), and the age at which study participants completed their education (#845.0.0). Lastly, five cognitive measures were included in our analysis: fluid intelligence (#20191.0.0), the duration to complete the numeric path in the trail making task (#6348.2.0), the duration to complete the alphanumeric path in the trail making task (#6350.2.0), the number of correct digit matches (#20159.0.0), and the number of correct pattern completions (#6373.2.0). During our experiments, the following values were considered abnormal records and discarded: age when education was completed < 15 and stiffness index > 3.

### 2.6. Bias detection

The association between the functional biomarker μ and study participants’ ethnicity was investigated as follows. UKBB study participants belong to different ethnic groups, which were reported as follows. Three large categories were defined: Asian, Black, and White. The Asian category was further divided into Bangladeshi, Chinese, Indian, and Pakistani. The Black category into African and Caribbean, and the White category into British and Irish. Study participants can belong to several groups at the same time or no group at all when data is missing. Since the Bangladeshi group in our sample was made of only six people, the subgroup was discarded during the statistical tests: Bangladeshi were only considered as part of the Asian category. In this work, the ethnic categories and the ethnic groups were recovered and encoded into binary values. Kruskal–Wallis H tests (one-way ANOVA on ranks) were then conducted to establish whether the categories and the groups were associated with significantly different functional aging biomarker values. The statistical tests were conducted independently for men and women to avoid introducing sex-related biases into the analysis.

For MESA study participants, four proportions could be recovered for each individual: the proportion of Hispanics in the neighborhood where the study participants were living at the time of the scan, the proportion of non-Hispanic Whites living in this neighborhood, the proportion of non-Hispanic Blacks, and the proportion of Asian Americans living in the area. Two statistical tests were used to investigate potential biases: first, the Pearson correlations between each proportion and the measure μ were reported. Then, each individual was assigned to the most frequent group in the area, and the Kruskal–Wallis H tests described in the previous paragraph were repeated (4 groups). Once again, these two statistical tests were conducted separately for men and women.

### 2.7. Interpreting the age predictors

Nonlinear SVR models cannot be interpreted directly (Cortes, 1995; Smola and Schölkopf, 2004; Drucker et al., 1996), so this section will focus on linear age regressors. These regressors indicate a direction, de-noted β in Eq. (9), that summarizes the aging effects on brain function in the most accurate way to predict the age of study participants. The direction β obtained for the best linear age predictors was projected using the truncated SVD basis W to obtain an upper triangular matrix, which was completed into a square symmetric matrix ℬ. In parallel, an average connectome 𝒜 was calculated. The component-wise multiplication between these matrices was calculated to distinguish two types of connections: first, the pairwise connections between brain regions that are reinforced by aging, either because they correspond to positive correlations that further increase with age or to anti-correlations that further decrease with age, and second, the connections that weaken with age.

To aid interpretation of these matrices, the two hemispheres were considered separately, and cortical ROIs located within the same large HCP atlas regions were grouped by averaging corresponding values. This operation yields matrices of size 22×22 (Glasser et al., 2016). The average 22×22 connectomes were directly reported, while the 22×22 aging directions were normalized to a unit standard deviation to improve their readability. Bures–Wasserstein average connectomes 𝒜 were transformed into covariance matrices C(𝒜) by reverting the Bures–Wasserstein transform as follows:

(14) $$C(𝒜)={\left(\frac{1}{2}𝒜+I\right)}^{2}$$

(15) $$=\frac{1}{4}{𝒜}^{2}+𝒜+I$$

Since a small variation of amplitude δ around 𝒜 in the direction ℬ produces the covariance matrix:

$$C\left(𝒜+\delta ℬ\right)={\left(\frac{1}{2}𝒜+\frac{\delta }{2}ℬ+I\right)}^{2}\;=C\left(𝒜\right)+\frac{\delta^{2}}{4}{ℬ}^{2}\;+\delta \left[ℬ+\frac{1}{4}𝒜ℬ+\frac{1}{4}ℬ𝒜\right]$$

The following matrix was considered when interpreting the connectivity changes with age modeled by the BW linear models:

(16) $$D(ℬ)=ℬ+\frac{1}{4}𝒜ℬ+\frac{1}{4}ℬ𝒜$$

This matrix captures the connectivity changes associated with aging among study participants near the average age. For larger age variations, it would have been possible, as a valid alternative approach not tested in this work, to predict Bures–Wasserstein connectomes for various ages and then transform the predicted BW connectomes into covariance matrices like the average connectome.

## 3. Experimental results

### 3.1. Parameter setting

**Fig. 3.**
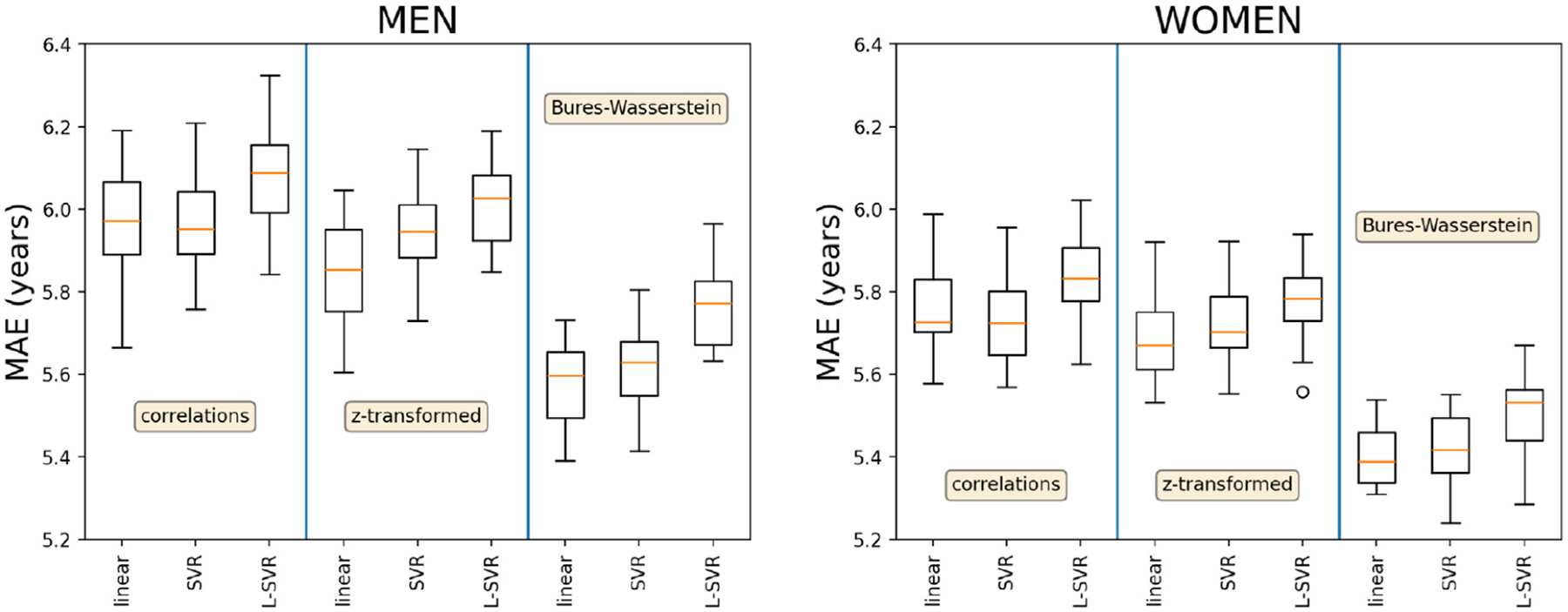
**Mean absolute errors** (MAE) of the age predictions obtained during the Monte Carlo cross-validation conducted to set the parameters of the age predictors. **First row:** MAE as a function of the variance retained by the truncated SVD, for the Bures–Wasserstein log connectomes $E_{s}$ and best ridge and SVR parameters. **Second row:** overall best nine age predictors.

Fig. 3 presents the results obtained during the Monte Carlo cross-validation of the age predictors. The first row illustrates the effects of the truncated SVD on the age predictors derived for the Bures–Wasserstein log connectomes $E_{s}$. The second row reports the overall best predictions and demonstrates the effect of the connectome transforms and the choice of machine learning model. Better age predictions were obtained for women, for all age predictors, but the results obtained in both groups supported the same conclusions. Bures–Wasserstein log connectomes produced significantly better results. In this sample, ridge regressions and SVR were also more robust than linear SVR models (p-value 5.96×10^−7^ Wilcoxon signed-rank test for the pairwise comparison between ridge regression and linear SVR, 1.97 × 10^−6^ when comparing SVR and linear SVR, no significant difference between ridge regression and SVR).

### 3.2. Methods validation

**Fig. 4.**
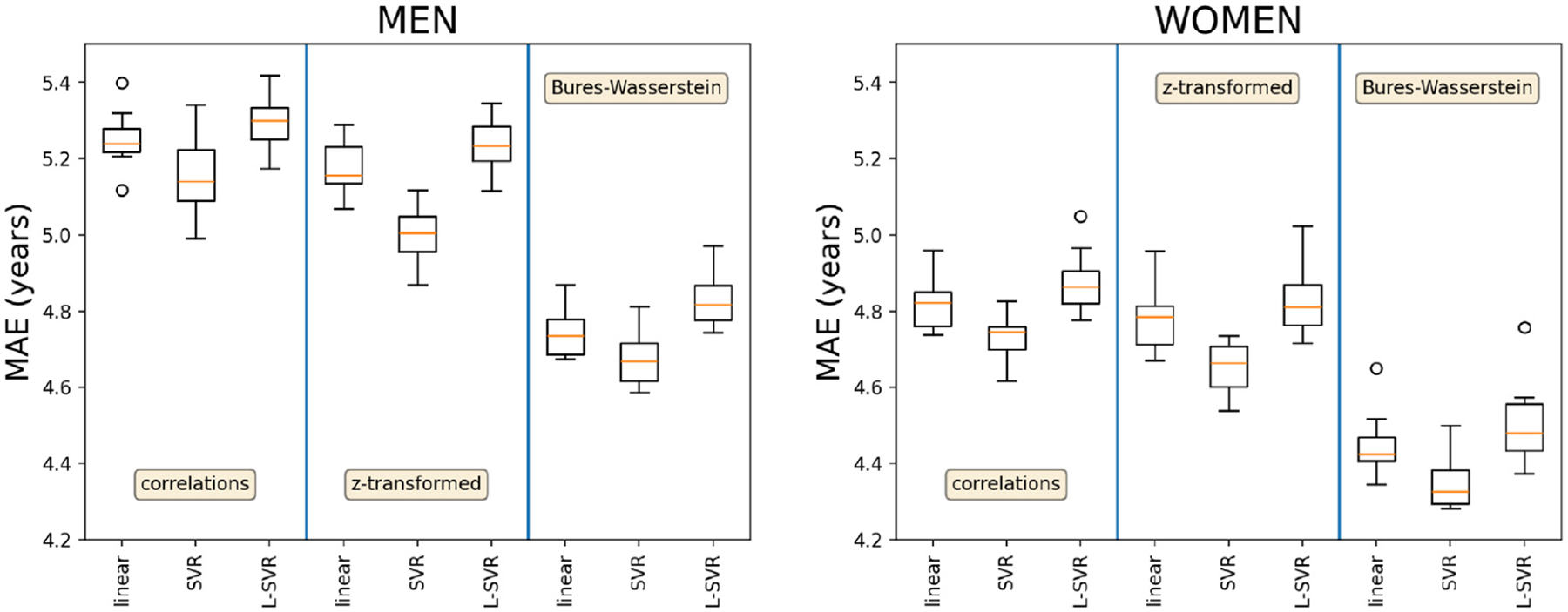
Ten-fold cross-validation age prediction mean absolute errors (MAE).

Fig. 4 presents the final ten-fold cross-validation results. Once again, better age predictions were obtained for women and the Bures–Wasserstein log connectomes. However, SVR predictors consistently outperformed linear and L-SVR predictors. For the Bures–Wasserstein connectomes producing the best results, SVR predictors significantly outperformed linear predictors (Wilcoxon signed-rank test p-value of 0.0020) and L-SVR predictors (p-value 0.0020 Wilcoxon signed-rank test). Similarly, for men, SVR predictors achieved significantly better predictions than linear predictors (p-value 0.0059 Wilcoxon signed-rank test) and Linear SVRs (p=0.0020 Wilcoxon signed-rank test).

### 3.3. Brain aging biomarkers validation

Table 3 presents the associations between UKBB biomarkers and the functional brain aging biomarker μ derived from the most reliable age predictor, the age predictor relying on support vector regression. All the biomarker values released by the UK Biobank were included in our analysis. BMI, number of illnesses and treatments, processed meat and alcohol consumption, and the overall health measure could be recovered for more than 99.8% of the UKBB study participants with processed rsfMRI data. Blood pressure and pulse rate could be recovered for 92.96% of the study participants considered in our work, but arterial stiffness could be calculated for only 33.35% of the people. The level of education was obtained for 52.3%, the correct number of digits and patterns for 51.6% and 65.79%, respectively, and the trail-making task was completed by 66.49% of the study participants with fMRI data. These results indicate that the linear correction successfully removed the association between μ and the age at scan (UKBB #21003.2.0). Most of the biomarkers present a very significant association with our brain aging biomarker. Whether significant or not, all the associations are in the expected directions. For instance, the functional brain aging biomarker μ tends to decrease when the body mass index, the number of treatments, and the blood pressure increase. On the other hand, a smaller alcohol consumption and a better overall health correspond to significantly larger μ values. All cognitive measures are significantly associated with μ and indicate that higher μ correlates with greater fluid intelligence, better scores on digit-matching and pattern-completion tasks, and shorter trail-making times. However, none of the Pearson correlations reached an absolute value larger than 0.1, indicating that, despite their significance, the associations are weak and obscured by ample noise. The results presented in the supplementary Section 1 establish that the brain aging biomarkers derived from the other age predictors are also significantly associated with the UKBB biomarkers, and in the same directions.

### 3.4. Ethnicity-related biases

The UKBB cohort considered in this work consists of 564 Asians (315 men, 249 women), 293 Blacks (121 men, 172 women), and 36,836 Whites (17,230 men, 19,606 women), including 116 individuals belonging to more than one category. The cohort consist of 111 Chinese (43 men, 68 women), 271 Indian (166 men, 105 women), 60 Pakistani (43 men, 17 women), 167 Caribbean (57 men, 110 women), 124 African (62 men, 62 women), 34,587 British (16,269 men, 18,318 women), and 975 Irish (478 men, 497 women). The four ethnic groups defined in MESA are the following: 221 Hispanics (97 men, 124 women), 548 non-Hispanic Whites (267 men, 281 women), 203 non-Hispanic Blacks (90 men, 113 women), and 85 Asians (42 men, 43 women). As shown in Table 4, Kruskal–Wallis H tests detected no group differences in the UKBB. Similarly, the four MESA ethnic groups present brain aging biomarkers that are not significantly different, and correlation tests do not detect significant associations between the ethnic composition of the neighborhood and our brain aging biomarkers. These reassuring results suggest that our fMRI measures are not biased by participants’ ethnicity.

### 3.5. Brain aging

**Fig. 5.**
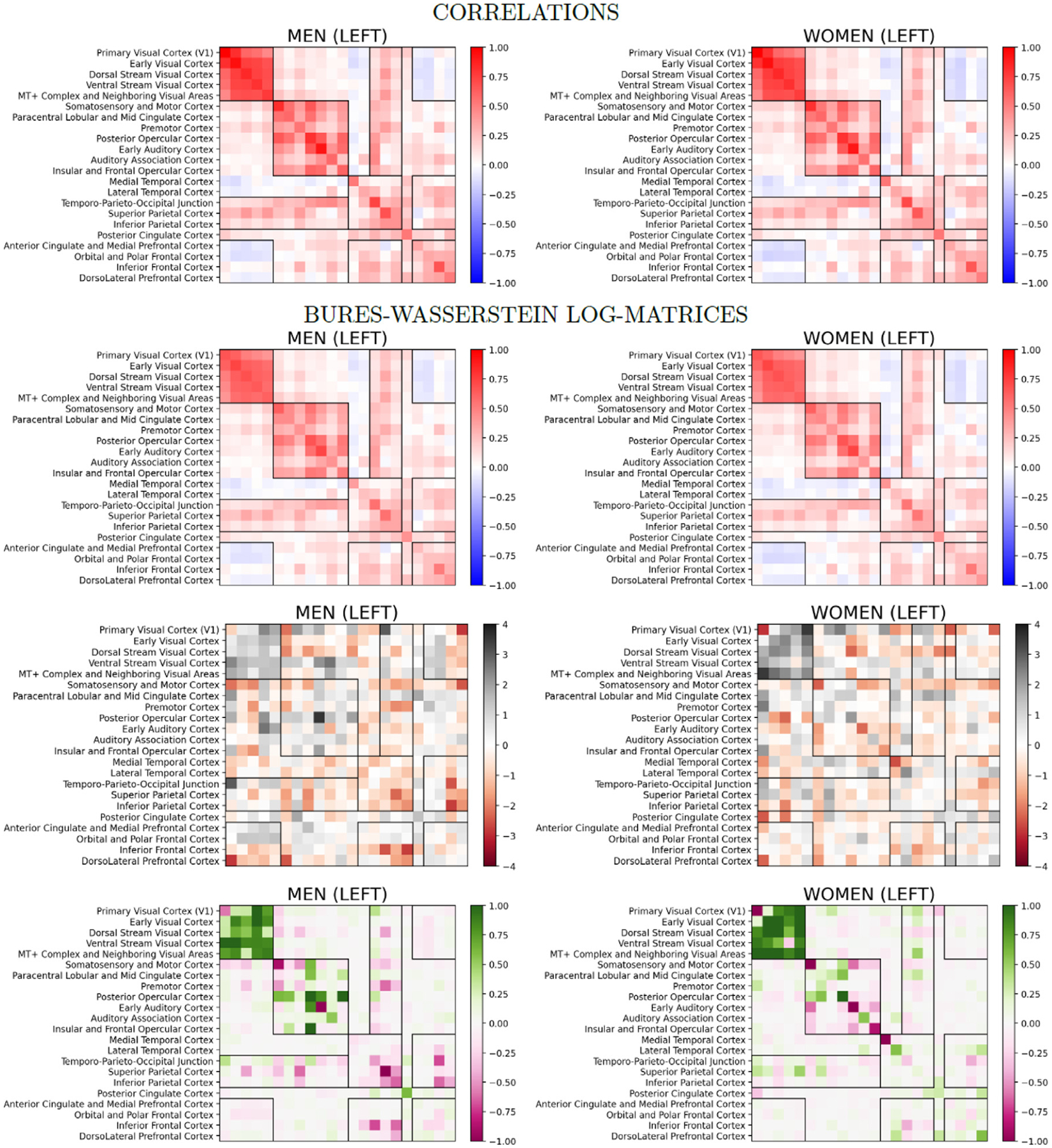
For the left hemisphere and separately for men and women: average correlation connectome (top row), covariance matrix derived from the average Bures–Wasserstein connectomes (C(𝒜), second row), aging direction selected by the BW linear model (D(𝒜), third row), and the product between average and direction indicating connectivity strengthening/weakening.

Fig. 5 reports the mean connectomes derived from the Bures–Wasserstein connectomes for the left hemisphere, and the connectivity changes predicted by the linear BW aging model for this brain hemisphere. First, the results indicate a great similarity between the average BW connectomes transformed into a covariance matrix C(𝒜) and the average connectome derived from the original correlations, which validates the use of the inverse BW transform. Then, we observe similar aging effects captured by the age predictors independently derived for men and women (Spearman correlation between the effects: $\rho =0.545\;p<10^{-38}$, between the products between aging effects and average connectomes: $\rho =0.474\;p<10^{-27}$). These changes correspond to a strengthened connectivity inside the visual cortex, except within V1, a weakened connectivity in the somatosensory and motor cortex, and between parietal regions and the rest of the brain, except V1. For men, the connectivity between parietal regions is also strongly weaker with age, while this effect is not present for women. A weakening of the functional connectivity inside the medial temporal cortex is noticeable in both groups. Connectivity changes for the anterior cingulate cortex (ACC) used to extract the salience network (Seeley, 2019) and the posterior cingulate cortex (PCC) considered when investigating the default mode network (Buckner et al., 2008) are not noticeable, except for reinforced connectivity within the PCC. On the other hand, the central executive network (Menon, 2011) extracted by considering the connectivity of the dorsolateral prefrontal cortex (DLPFC) and/or the inferior frontal cortex present a marked decline in men, and a weak decline in women. Similar results are presented in the Supplementary Section 1 for the right hemisphere. We also note in this appendix that the aging models derived from the BW connectomes are more stable than the models derived from correlations or Fisher z-transformed correlations.

## 4. Discussion

### 4.1. Summary

In this work, we investigated how machine learning brain aging biomarkers could be derived from large sets of functional connectomes. More specifically, we trained machine learning models to predict the age of study participants from connectomes derived from their resting-state fMRI scans. We combined four large sets of functional MRI scans during our experiments to consider a total of 40,414 individual connectomes. We compared the performance of hundreds of age predictors, and we selected the best predictors to quantify a measure of accelerated aging. Our experiments indicated that this measure can be biased by ethnicity, so we investigated eleven approaches to remove these unwanted biases. Overall, the function brain age predictions obtained in this work, on average within four to five years of the real neuroimaging study participant ages, are on par with brain age predictions reported for similar datasets in the literature (Cole, 2020). For instance, a study conducted for the UK Biobank (Miller et al., 2016; Alfaro-Almagro et al., 2018; Sudlow et al., 2015) reported a mean absolute error close to four years for T1-weighted MRI age prediction (4.14 years), and slightly above five years for fMRI-based predictors (5.26 years) (Cole, 2020). We have shown here that fMRI-based age prediction can be improved to the point of closing most of this gap by reaching errors between 4.2 years for women and 4.5 years for men.

### 4.2. Connectome transforms

First, our experiments indicate that Bures–Wasserstein log-matrices (Honnorat et al., 2024) produce better age predictors than standard correlations and Fisher z-transformed correlations. We note that better harmonizations were already observed when using this transform in our prior work (Honnorat et al., 2024). We would hypothesize to explain the performance boost of machine learning models observed when using the Bures–Wasserstein transform that the transform generates log matrices that are not restrained into a small part of the vectorial space of the squared matrices, contrary to original connectomes that are restrained into the cone of the positive semidefinite matrices (Honnorat et al., 2024), and that this freedom helps statistical methods when they model a distribution of connectomes.

### 4.3. Best age predictors

During our experiments, we observed that the simple linear ridge regression age predictors produced the best predictions when fewer samples were available, such as during the parameter setting experiments that were conducted using nearly two thousand connectomes. The nonlinear Support Vector Regressors age predictors relying on Radial Basis Functions produced significantly better results during the method validation experiments conducted using nine times more data. We would interpret these results by concluding that a sample size of two thousand connectomes was not sufficient to prevent SVR overfitting despite the use of a truncated SVD to compress the data. The sensitivity analysis presented in supplementary Section 2 supports this interpretation by showing that the best nonlinear SVR age predictor selected during the parameter-setting phase outperforms the best linear age predictor only when the sample size is exceeds 3000. Based on these experimental results, we suggest collecting more than 3000 connectomes in future age-regression studies before exploring nonlinear frameworks, such as Support Vector Regression and deep learning models.

### 4.4. Brain health

Age predictions generated by machine learning statistical models can be considered like brain disease biomarkers when the diseases are known to induce brain damage similar to aging, for example, by causing significant cognitive decline or brain atrophy. These diseases appear as accelerated aging, with the brains of affected individuals looking older than they should according to statistical models (Cole et al., 2017, 2018; Cole, 2020; Moguilner et al., 2024). We have validated this approach once again for the UKBB dataset by measuring the association between a large panel of UKBB biomarkers and brain aging biomarkers derived from the age predictors proposed in this work. Our results reveal very significant associations supporting the use of machine learning models to track brain health: healthier UKBB participants tend to be predicted with a younger brain age than, for instance, participants suffering from multiple illnesses, who are at risk for cardiovascular diseases, and who are drinking more alcohol. Our results suggest, once again, the possibility of applying machine learning tools to medical images to define “biological clocks” tracking the health of human organs over the lifespan (Moguilner et al., 2024). That being said, the small amplitude of the associations observed, UKBB health biomarkers explaining usually less than a percent of the variance of our functional aging biomarker, calls for careful interpretations and future investigations. Our present work indicates that our functional aging biomarkers vary in the expected directions for health measures, but they are also capturing specific, and hopefully valuable, information not present in the basic UKBB health markers we have investigated.

### 4.5. Limitations

We have compared hundreds of age predictors in this work, but the large computational time required to carry out our complete experiments has limited the number of methods we could explore. Several additional approaches could not be tested. These interesting approaches to consider in future investigations include additional nonlinear machine learning age predictors (Ho, 1995; LeCun and Bengio, 1995), and other types of log-matrices (Fletcher and Joshi, 2007; Arsigny et al., 2006).

Despite the limited fraction of connectome variability attributable to dataset differences, reported in Supplementary Section 4, and the use of a SOTA harmonization procedure (Johnson et al., 2006; Fortin and Cullen, last checked in Nov. 2025), the preprocessing heterogeneity resulting from the derivation of the UK Biobank connectomes from ICA-denoised data (Pruim et al., 2015b,a) remains a limitation of this work, the unprecedented scale of the UKBB dataset preventing a costly reprocessing from scratch using the image processing pipeline applied to the other cohorts.

As reported in Section 3.4 and Table 4, statistical tests failed to detect significant ethnicity-related biases in the functional aging biomarkers we derived for the MESA and UK Biobank study participants. This result could indicate either that there are no disparities in the datasets or that disparities were erased during data processing or washed out by the inherent noise of fMRI scans. Additional investigations will be required to validate these hypotheses, assess ethnicity-related biases in a different way, and determine whether they are clinically relevant.

## 5. Conclusion

In this work, we explore the use of machine learning models to derive a biomarker of brain aging from the resting-state functional MRI scans of forty thousand study participants. We compare the effects of three functional connectome transformations, twelve data compression levels, and three families of age predictors. Two levels of cross-validations are conducted to gradually reveal the most accurate age predictors, which are finally used to build a set of functional aging biomarkers capturing the impact of aging on brain function. Our results indicate that a new connectome transform derived from the Riemannian geometry induced by the Bures–Wasserstein metric can significantly improve functional age predictions. We observed that two thousand connectomes could have an insufficient sample size to produce nonlinear age predictors better than basic linear models penalized by ridge penalties. We did not observe significant ethnicity-related biases in the set of UK Biobank study participants with resting-state fMRI data. It was the same for our MESA study participants. Lastly, we demonstrated how to interpret Bures–Wasserstein connectomes. We hope that our result will pave the way for more accurate and reliable biomarkers of brain aging that could be used to refine brain disease diagnosis and monitor the effects of neurological and psychiatric treatments.

## Supplementary Material

supplement

## Figures and Tables

**Table 1:** Study participants included in this work. Two-sample T-tests were conducted and the associated Cohen’s D effect sizes (d) reported to either compare the ages of men and women within a study (*) or to compare a study with the entire set of participants (^†^). Cohen’s D reported in bold font correspond to statistical tests significant at level 0.001.

DATA	FHS	HCP	MESA	UKBB	ALL
participants	413	1015	1057	37929	40414
men	211	471	496	17787	18965
women	202	544	561	20142	21449
mean age (std)	58.37 (10.35)	28.82 (3.58)	74.19 (8.16)	63.69 (7.54)	63.03 (9.48)
men	58.54 (10.35)	28.07 (3.61)	74.18 (8.07)	64.38 (7.65)	63.66 (9.66)
women	58.20 (10.16)	29.46 (3.43)	74.19 (8.23)	63.08 (7.39)	62.47 (9.28)
Cohen’s |d|	0.032^a^	**0.396** ^a^	0.001^a^	**0.172** ^a^	**0.126** ^a^
	**0.491** ^b^	**3.649** ^b^	**1.181** ^b^	**0.076** ^b^	

a age comparison between men and women within a study (two-sample T-test).

b age comparison between all the participants in a study and ALL (two-sample T-test).

**Table 2:** Data stratification. Parameter setting (PS) experiments were conducted using 1895 scans for men’s age predictors and 2144 for women’s age predictors. The final method validation (MV) was conducted using 17070 scans for men and 19305 scans for women. Total scan counts are indicated to recall the participant counts reported in Table 1.

scans	Men			Women			Total
STUDY	PS	MV	total	PS	MV	total	
FHS	21	190	211	20	182	202	413
HCP	47	424	471	54	490	544	1015
MESA	49	447	496	56	505	561	1057
UKBB	1778	16009	17787	2014	18128	20142	37929
**all PS**	**1895**			**2144**			
**all MV**		**17070**			**19305**		

PS: parameter setting, MV: method validation.

**Table 3:** Association between the best brain aging biomarkers μ and the 16 UKBB biomarkers: body mass index (BMI), number of illnesses (illness), number of treatments (treatments), overall health (health), systolic (sbp) and diastolic (dbp) blood pressures, arterial stiffness index (asi), age when education was completed (education), fluid intelligence (FI), duration of the numeric trail making (TMT 1), duration of the alphanumeric trail making (TMT 2), correct number of digit matches (digits) and pattern completions (patterns). β is also equal to the Pearson correlation between μ and the biomarkers.

biomarker	n	adjusted $R^{2}$	standardized β	95% CI		p-value
				min	max	
age	37 929	−2.64 × 10 ^−5^	2.41 × 10 ^−12^	−0.010	0.010	1
BMI	37 878	0.002682	−0.05204	−0.06210	−0.04198	3.89 × 10 ^−24^
illness	37 923	0.000943	−0.03113	−0.04119	−0.02107	1.33 × 10 ^−9^
treatments	37 923	0.002004	−0.04506	−0.05512	−0.03501	1.65 × 10 ^−18^
health	37 859	0.001453	0.03846	0.02839	0.04853	7.11 × 10 ^−14^
sbp	35 260	0.006181	−0.07880	−0.08920	−0.06839	1.11 × 10 ^−49^
dbp	35 260	0.005588	−0.07494	−0.08535	−0.06453	4.28 × 10 ^−45^
pulse rate	35 260	0.003081	−0.05576	−0.06618	−0.04534	1.09 × 10 ^−25^
asi	12 651	0.000563	−0.02534	−0.04277	−0.00792	0.0044
alcohol	37 911	0.000470	−0.02227	−0.03234	−0.01221	1.44 × 10 ^−5^
meat	37 895	2.19 × 10 ^−6^	−0.00535	−0.01541	0.004723	0.29
education	19 838	7.93 × 10 ^−5^	0.01139	−0.00253	0.02531	0.11
FI	20 176	0.001199	0.03534	0.02155	0.04913	5.14 × 10 ^−7^
TMT 1	25 219	0.000472	−0.02262	−0.03496	−0.01028	0.00033
TMT 2	25 219	0.000647	−0.02620	−0.03854	−0.01386	3.16 × 10 ^−5^
digits	19 587	0.000877	0.03047	0.01647	0.04447	2.00 × 10 ^−5^
patterns	24 953	0.001849	0.04347	0.03107	0.05587	6.47 × 10 ^−12^

**Table 4:** P-values reported for the statistical tests conducted to assess the presence of ethnicity-related biases. None of the Kruskal–Wallis test detected group differences. For MESA, none of the correlations between the proportions of Hispanics (H), non-Hispanic Whites (NHW), non-Hispanic Whites (NHB), or Asians (A) living in the neighborhood and the brain aging biomarkers was significant.

Statistical test p-values	MEN	WOMEN
UKBB (3 categories)	0.1277^a^	0.3453^a^
UKBB (7 groups)	0.1101^a^	0.6825^a^
MESA (4 groups)	0.8004^a^	0.9310^a^
MESA (4 proportions)		
H	0.5509^b^	0.9120^b^
NHW	0.6712^b^	0.5164^b^
NHB	0.3804^b^	0.8771^b^
A	0.8186^b^	0.3925^b^

a Kruskal–Wallis H.

b Pearson correlation test.

## Data Availability

FHS data access procedures can be obtained here: https://www.framinghamheartstudy.org/fhs-for-researchers/. The Human Connectome Project minimally preprocessed young adult dataset imaging data and associated NIH Toolbox measures are publicly available at https://db.humanconnectome.org/. MESA consortium can be contacted via the following website: https://www.mesa-nhlbi.org/. The UKBB data mentioned in this work is available to all researchers and can be accessed upon approval of the UK Biobank (https://www.ukbiobank.ac.uk/enable-your-research/apply-for-access).

## Appendix A. Supplementary data

Supplementary material related to this article can be found online at https://doi.org/10.1016/j.brainresbull.2026.111815.
