# Supplementary material for: Derivation of machine learning brain aging biomarkers for a set of forty thousand functional connectomes
Source: Brain Res Bull. Author manuscript; Available in PMC 2026 May 26. (PMC13202619; doi:10.1016/j.brainresbull.2026.111815)
Supplement: supplement [file NIHMS2166101-supplement-supplement.pdf]

Derivation of Machine Learning Brain Aging Biomarkers for a Set of Forty Thousand Functional Connectomes

Nicolas Honnorat, Di Wang, Ngoc-Huynh Ho, David Martinez,
Sachintha Ransara Brandigampala, Susan R. Heckbert, Mohsen Bahrami,
Jayandra Jung Himali, Charlie DeCarli, Alexa Beiser, Timothy M. Hughes,
Sudha Seshadri, Mohamad Habes

1 Brain Aging

Figure 1 indicates that the mean connectomes and aging effects reported in the main document for the left hemisphere are similar to the effects observed for the right hemisphere. Figure 2 also indicates that the aging effects reported for the BW connectomes are more stable than the effects discovered using correlations and Fisher z-transformed correlations.

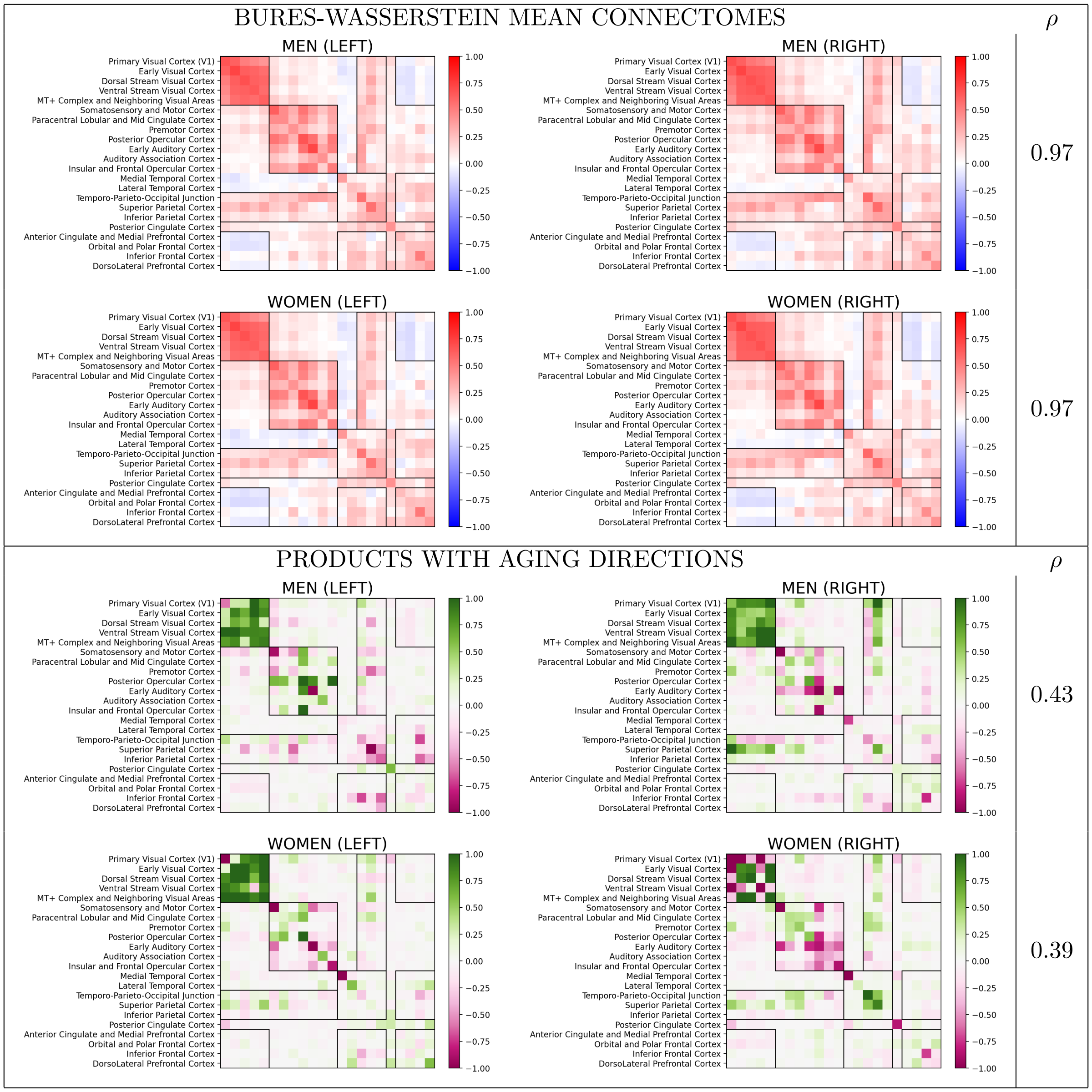

Figure 1: Comparison between the BW connectomes and aging effects obtained for the left and the right hemispheres. All the Spearman correlations ρ were significant with $p < 10^{-18}$.

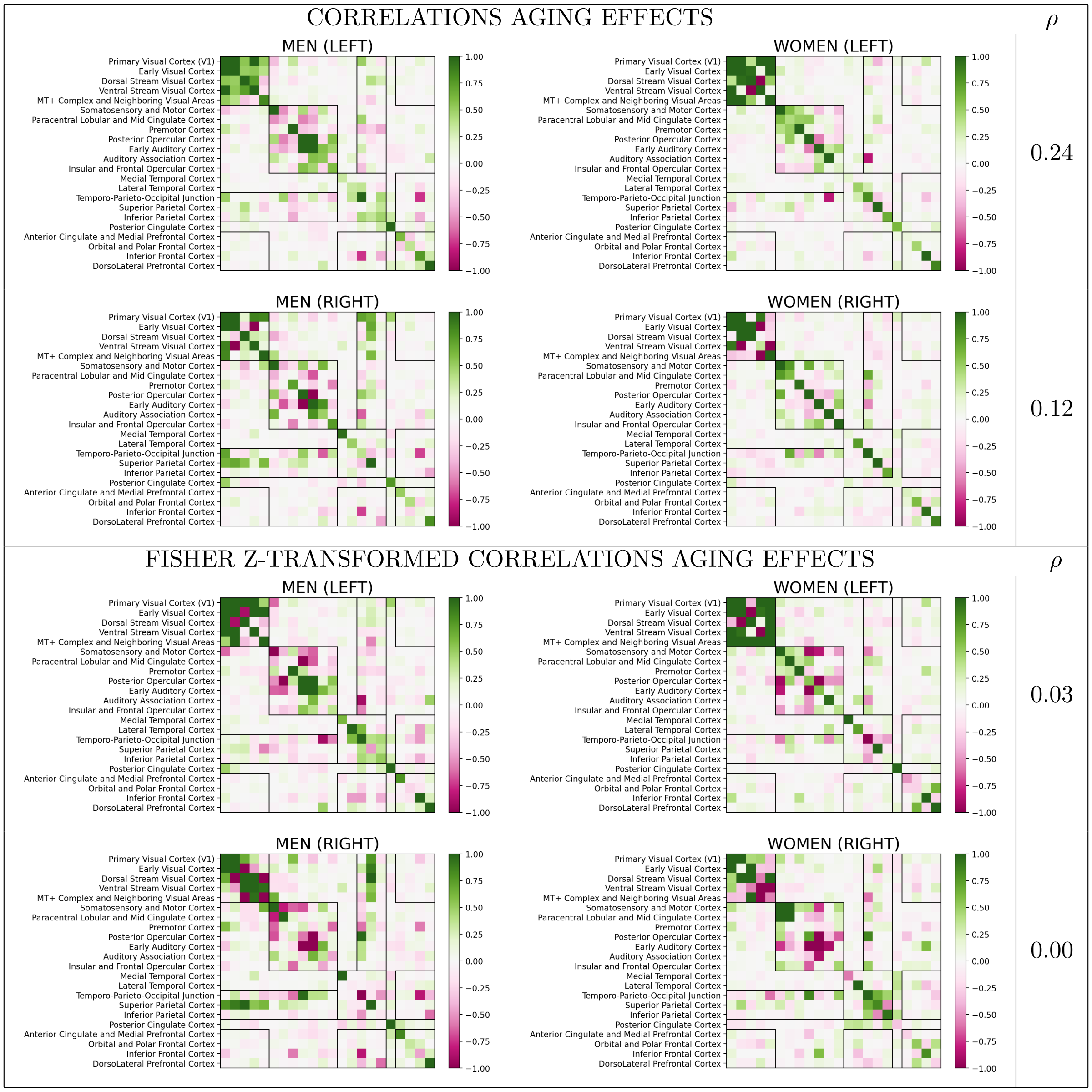

Figure 2: Comparison between the aging effects obtained for men and women for the correlations and Fizher z-transformed correlations. The Spearman correlations ρ obtained for the correlations were significant, with respectively $p = 6.15{-}8$ and $p = 0.0086$ for the left and right hemisphere, respectively. On the other hand, the aging models derived for Fisher z-transformed correlations selected aging directions that were uncorrelated (both $p > 0.48$).

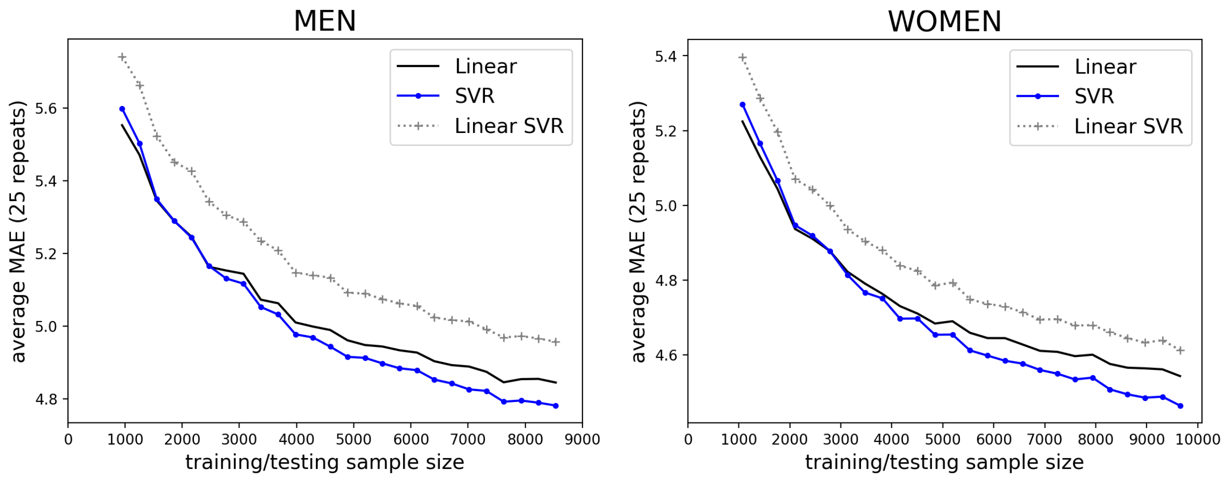

Figure 3: Median absolute error (MAE) as a function of the sample size for the best three age predictors.

2 Sample Size Sensitivity Analysis

A sensitivity analysis was conducted to investigate the accuracy of the age predictors as a function of the number of connectomes used to train the models. During this analysis, for men and women separately, 25 sample sizes ranging from 5% to 45% of the total number of harmonized Bures-Wasserstein log-matrices were calculated. For each sample size, two non-overlapping sets of connectomes were randomly selected to train and test the best linear, SVR, and linear-SVR age predictors. The procedure was repeated 25 times, and the 25 test MAEs for each age predictor, sex group, and sample size were averaged. The results, presented in Figure 3, suggest that linear models are more robust for sample sizes smaller than 3,000. For larger sample sizes, the nonlinear SVR gradually becomes the best model.

biomarker	n	adjusted R^2	standardized β	95% CI		p-value
				min	max	
age	17787	-5.62×10^{-5}	-2.44×10^{-11}	-0.0147	0.0147	1
BMI	17765	0.005770	-0.07633	-0.09099	-0.06166	2.26×10^{-24}
illness	17784	0.001507	-0.03954	-0.05423	-0.02485	1.33×10^{-7}
treatments	17784	0.003705	-0.06132	-0.07600	-0.04665	2.72×10^{-16}
health	17762	0.003181	0.05689	0.04221	0.07158	3.25×10^{-14}
sbp	16583	0.006230	-0.07931	-0.09448	-0.06413	1.48×10^{-24}
dbp	16583	0.005141	-0.07212	-0.08730	-0.05693	1.43×10^{-20}
pulse rate	16583	0.003806	-0.06218	-0.07737	-0.04699	1.11×10^{-15}
asi	6058	0.000326	-0.02216	-0.04734	0.003025	0.085
alcohol	17779	0.000731	-0.02806	-0.04276	-0.01337	0.000182
meat	17774	0.000339	-0.01989	-0.03459	-0.005190	0.008012
education	8968	6.67×10^{-5}	0.01335	-0.00735	0.03405	0.21
FI	9198	0.000819	0.03046	0.01003	0.05089	0.0035
TMT 1	11825	0.000566	-0.02550	-0.04352	-0.00748	0.0055
TMT 2	11825	0.000664	-0.02736	-0.04538	-0.00934	0.0029
digits	8905	0.002289	0.04900	0.02825	0.06975	3.73×10^{-6}
patterns	11704	0.001345	0.03781	0.01971	0.05592	4.28×10^{-5}

Table 1: For **men**, association between the best brain health measure μ and the 16 UKBB biomarkers: body mass index (BMI), number of illnesses (illness), number of treatments (treatments), overall health (health), systolic (sbp) and diastolic (dbp) blood pressures, arterial stiffness index (asi), age when education was completed (education), fluid intelligence (FI), duration of the numeric trail making (TMT 1), duration of the alphanumeric trail making (TMT 2), correct number of digit matches (digits) and pattern completions (patterns). β is also equal to the Pearson correlation between μ and the biomarkers.

3 Brain Health Measure and Sex Biases

The association between the brain health measure μ and the UKBB biomarkers were re-calculated after focusing either on men or women to investigate the presence of sex-related biases in the brain health measure. The results reported in Table 1 and Table 2 demonstrate that very similar associations are observed in both age groups. These effects are also close to effects reported in the main document for the whole group. These observations suggest that if sex-related biases are present they are too small to alter our conclusions.

biomarker	n	adjusted R^2	standardized β	95% CI		p-value
				min	max	
age	20142	-4.97×10^{-5}	2.89×10^{-11}	-0.01381	0.01381	1
BMI	20113	0.001165	-0.03485	-0.04867	-0.02104	7.66×10^{-7}
illness	20139	0.000529	-0.02406	-0.03787	-0.01025	0.00064
treatments	20139	0.000928	-0.03127	-0.04508	-0.01747	9.06×10^{-6}
health	20097	0.000401	0.02122	0.00740	0.03505	0.0026
sbp	18677	0.006631	-0.08176	-0.09605	-0.06746	4.50×10^{-29}
dbp	18677	0.006456	-0.08068	-0.09498	-0.06638	2.36×10^{-28}
pulse rate	18677	0.002420	-0.04974	-0.06406	-0.03541	1.04×10^{-11}
asi	6593	0.000664	-0.02857	-0.05270	-0.00443	0.0020
alcohol	20132	0.000275	-0.01802	-0.03183	-0.00421	0.011
meat	20121	2.05×10^{-5}	0.00838	-0.00544	0.02220	0.23
education	10870	3.20×10^{-7}	0.00961	-0.00919	0.02841	0.32
FI	10978	0.001533	0.04029	0.02160	0.05899	2.41×10^{-5}
TMT 1	13394	0.000323	-0.01995	-0.03689	-0.00302	0.021
TMT 2	13394	0.000553	-0.02505	-0.04198	-0.00811	0.0037
digits	10682	0.000141	0.01531	-0.0037	0.03427	0.11
patterns	13249	0.002344	0.04919	0.03218	0.0662	1.47×10^{-8}

Table 2: For **women**, association between the best brain health measure μ and the 16 UKBB biomarkers: body mass index (BMI), number of illnesses (illness), number of treatments (treatments), overall health (health), systolic (sbp) and diastolic (dbp) blood pressures, arterial stiffness index (asi), age when education was completed (education), fluid intelligence (FI), duration of the numeric trail making (TMT 1), duration of the alphanumeric trail making (TMT 2), correct number of digit matches (digits) and pattern completions (patterns). β is also equal to the Pearson correlation between μ and the biomarkers.

4 Harmonization

The effectiveness of our harmonization procedure was validated by comparing the variance in the truncated SVD components of the transformed connectomes attributable to cohort differences before and after harmonization. For each component y, this variance was measured in two ways. First, by fitting the following linear model, where the binary variable 1_X was equal to 1 for the scans of cohort X and 0 otherwise:

$$y = \alpha + \beta 1_{FHS} + \gamma 1_{HCP} + \delta 1_{MESA} \tag{1}$$

and reporting the adjusted R^2 value associated with the linear model. And then, by fitting the same linear model to a residual obtained by removing linear age and sex effects from y. Adjusted R^2 values smaller than 10^{-6} were set to 10^{-6}. The results in Figure 4 indicate that, for almost all SVD components, the fraction of variance attributable to cohort differences was less than 1% before harmonization. The harmonization reduced the fraction of variance attributable to cohort differences in the residuals to adjusted R^2 values of less than 10^{-6} for almost all SVD components. As a result, even the fractions of variance including age and sex effects were reduced for most SVD components. Harmonization had very similar effects for the three types of connectomes. These results demonstrate the effectiveness of our harmonization procedure.

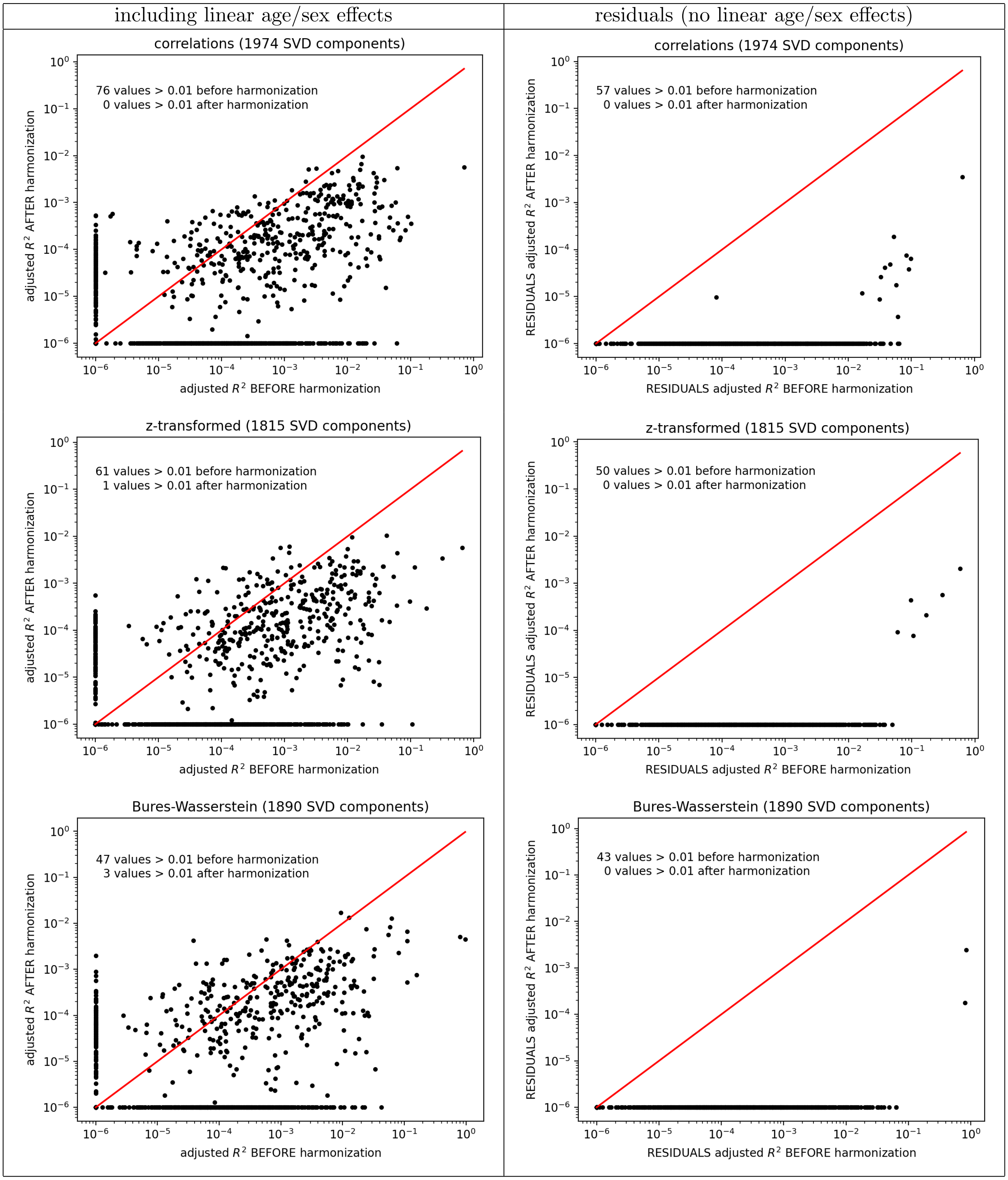

Figure 4: Comparison of the proportion of truncated SVD component variance attributable to cohort differences before and after harmonization, for the three type of connectomes, and with or without linearly removing age and sex effects.
